# Temperature increase altered *Daphnia* community structure in artificially heated lakes: a potential scenario for a warmer future

**DOI:** 10.1038/s41598-020-70294-6

**Published:** 2020-08-18

**Authors:** Marcin K. Dziuba, Magdalena Herdegen-Radwan, Estera Pluta, Łukasz Wejnerowski, Witold Szczuciński, Slawek Cerbin

**Affiliations:** 1 Faculty of Biology, Institute of Environmental Biology, Department of Hydrobiology, Adam Mickiewicz University in Poznań, Uniwersytetu Poznańskiego 6, 61-614 Poznan, Poland; 2 Department of Ecosystem Research, Leibniz-Institute of Freshwater Ecology and Inland Fisheries, Müggelseedamm 310, 12587 Berlin, Germany; 3 Faculty of Biology, Institute of Environmental Biology, Department of Behavioral Ecology, Adam Mickiewicz University in Poznań, Uniwersytetu Poznańskiego 6, 61-614 Poznan, Poland; 4 Institute of Geology, Geohazards Research Unit, Adam Mickiewicz University in Poznań, Krygowskiego 12, 61-680 Poznan, Poland

**Keywords:** Climate-change ecology, Community ecology, Freshwater ecology, Molecular ecology, Palaeoecology, Population dynamics

## Abstract

Under conditions of global warming, organisms are expected to track their thermal preferences, invading new habitats at higher latitudes and altitudes and altering the structure of local communities. To fend off potential invaders, indigenous communities/populations will have to rapidly adapt to the increase in temperature. In this study, we tested if decades of artificial water heating changed the structure of communities and populations of the *Daphnia longispina* species complex. We compared the species composition of contemporary *Daphnia* communities inhabiting five lakes heated by power plants and four non-heated control lakes. The heated lakes are ca. 3–4 °C warmer, as all lakes are expected to be by 2100 according to climate change forecasts. We also genotyped subfossil resting eggs to describe past shifts in *Daphnia* community structure that were induced by lake heating. Both approaches revealed a rapid replacement of indigenous *D. longispina* and *D. cucullata* by invader *D. galeata* immediately after the onset of heating, followed by a gradual recovery of the *D. cucullata* population. Our findings clearly indicate that, in response to global warming, community restructuring may occur faster than evolutionary adaptation. The eventual recolonisation by *D. cucullata* indicates that adaptation to novel conditions can be time-lagged, and suggests that the long-term consequences of ecosystem disturbance may differ from short-term observations.

## Introduction

Temperature increase, a major component of climate change^1^, is expected to exert a strong direct impact on the functioning of freshwater organisms, e.g., modifying their physiology, development, and/or fitness^2–5^. Rising temperatures will also trigger a multitude of indirect effects on aquatic habitats, including alteration of abiotic parameters (e.g., oxygen saturation and ice cover duration)^6,7^ and biotic interactions (e.g., changes in primary production, predation intensity, mismatches in the occurrence of interdependent species, and altered host-parasite interaction dynamics)^6,8,9^. These changes will have an impact on the performance of individuals, and consequently on the structure and functioning of populations and communities^10,11^. Based on the current effects of climate change and models of further warming, it is predicted that many species could be threatened with extinction^12,13^. Thus, forecasting species’ responses to the warming climate is a timely issue in ecology, evolutionary biology, and environmental protection.

According to Bellard and coauthors^14^, there are three non-exclusive types of organismal responses to climate change, namely, alterations in (1) time of occurrence (e.g., shifts in phenology), (2) space (e.g., altered distribution range), and (3) self (i.e., changes in organisms’ physiology, not related to spatial or temporal changes). A change in ‘self’ might avert the necessity for shifts in phenology or spatial range. However, an organism’s capacity for adaptation is constrained by physiological limitations on resistance to thermal extremes and a restricted capacity for evolution^15–17^. Species migrations and shifts in phenology that track favourable thermal conditions seem to be likelier and easier, and thus might be more frequent outcomes than evolutionary adaptation. It is expected that, in seeking thermal refuge or following optimal thermal conditions, many taxa will undergo distribution shifts towards higher latitudes and altitudes, where they will often become alien invaders^18,19^. Adaptation might enable indigenous species or populations to fend off potential invaders (e.g.,^20^). However, as warming increases, the success of invaders is expected to as well^21,22^.

In temperate freshwater communities, climate warming increases the risk of invasion that can ultimately lead to community restructuring^23–26^. There is abundant evidence that the community structure of the model organism for temperate lakes—the planktonic crustacean *Daphnia*—is shaped by variations among taxa in sensitivity to environmental constraints. Studies conducted on communities of the *Daphnia longispina* complex—a group of hybridizing species widespread in European lakes (e.g.,^27,28^)—have established that temperature is one of the major factors shaping the species composition of *Daphnia* communities. Specifically, *Daphnia galeata* inhabits warmer lakes than *D. longispina*^27^. Moreover, *D. galeata* and its hybrids have a greater propensity to actively overwinter (using parthenogenetic reproduction instead of diapause), and hence have a competitive advantage over *D. longispina* during mild ice-free winters^29^. These results suggest that an increase in temperature should favour *D. galeata* over other taxa, in particular *D. longispina*. However, it remains an open question whether such a pattern can simply be extrapolated to climate change forecasts.

Gradual increases in temperature can promote the establishment of pre-adapted invaders but may also induce evolutionary adaptations in native populations^30^ that increase their resistance to invasion^20^. The ultimate outcome will depend on which of these responses is more rapid and effective. So far, this remains a puzzle, which will require decades of monitoring of gradually warming ecosystems to solve. In the present study, we make use of an unprecedented opportunity to investigate a model of climate change in an ecosystem that has already experienced warming for over five decades. This system is composed of five natural lakes that are heated by power plants, in which the water temperature has increased by, on average, ca. 3–4 °C in comparison to non-heated (control) lakes nearby. Thus, the conditions in these lakes almost perfectly correspond to climate change predictions for the end of the twenty-first century^1^. We used this system of heated lakes, with *Daphnia* as a model organism, to investigate the impact of climate change on aquatic communities. We applied a threefold approach:

1. First, using microsatellite markers and mtDNA barcoding, we determined whether the distribution of *Daphnia* species from the *D. longispina* complex (i.e. *D. galeata*, *D. longispina*, and *D. cucullata*) differed between heated and non-heated lakes (‘change of space’ hypothesis). We compared the species composition of *Daphnia* communities in heated and control lakes, as well as the relative abundance of each of the three species within individual lakes. Our expectation was that *D. galeata* would demonstrate an advantage over *D. longispina* in heated lakes.
2. Second, we tested the ‘change of self’ hypothesis, also using microsatellite markers. If increased temperature in the heated lakes induced rapid adaptation, we would expect gene flow to decrease and genetic structure to arise among conspecific *Daphnia* populations from heated and control lakes. The discovery of such a pattern would serve as evidence for warming-mediated diversification between populations in heated and control lakes.
3. Third, we explored the historical records for changes in taxonomic composition. Using sediment cores representing the last few decades, we compared the bank of sexual resting eggs from one of the heated and one of the control lakes. We counted and measured ephippia (the structures that contain the sexual resting eggs), expecting that *Daphnia *would reduce the frequency of sexual reproduction and the production of resting eggs under warming conditions. We further screened the resting eggs from the heated lake using microsatellite markers, and used these data to analyse the community structure of *Daphnia* species before and after the onset of heating, in order to identify any changes induced by heating.

## Results

### Contemporary populations

**Figure 1:**
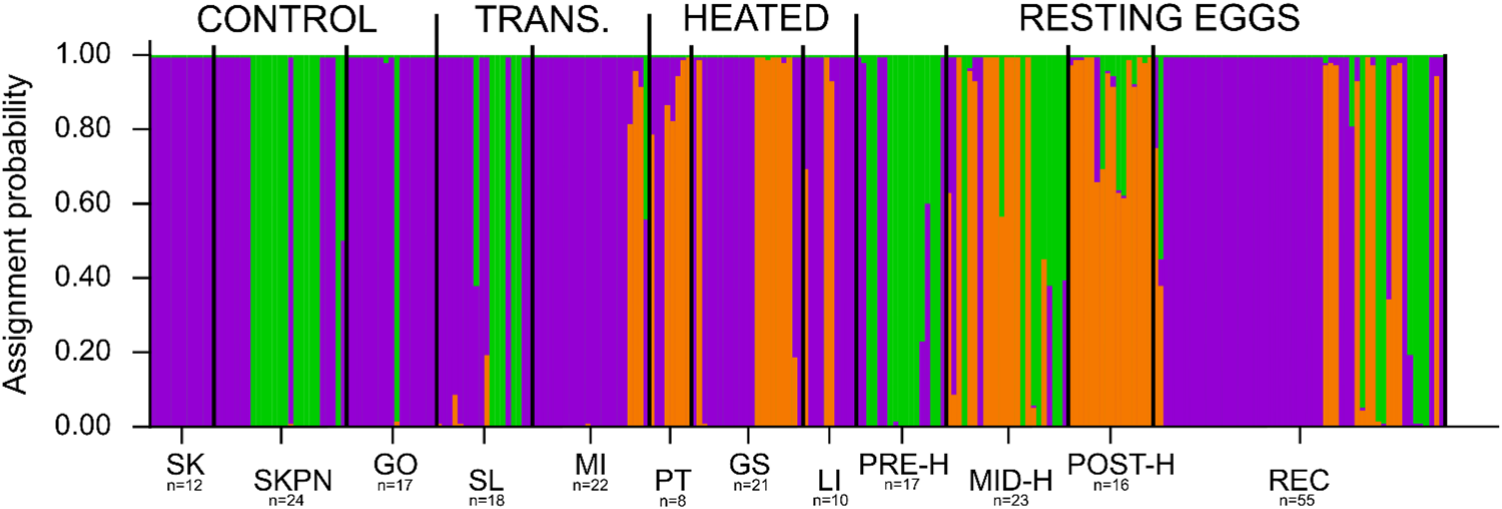
Plot of the assignment probability of contemporary samples and resting eggs to three genetically distinct clusters (different colours), inferred with STRUCTURE based on microsatellite data. Individuals are grouped by lake of origin. Each vertical line (see Supplementary Fig. S4 online for an individual-based version of the plot) represents the assignment probability of one individual to three inferred clusters (purple, green, or orange). Contemporary samples are grouped as follows: three control lakes (SK, SKPN, GO), two transitional (TRANS.) heated lakes (SL, MI), and three heated lakes (PT, GS, LI). Resting eggs are grouped as follows: PRE-H—produced before the onset of heating, MID-H—produced after the launch of the first plant but before the launch of the second power plant, POST-H—produced in the ca. 15 years following the launch of the second power plant, REC—produced recently, i.e. within 15 years of core collection. The n-value indicates sample size for each lake. Cluster colours correspond to those in Fig. 2 by similarity in composition.

**Figure 2:**
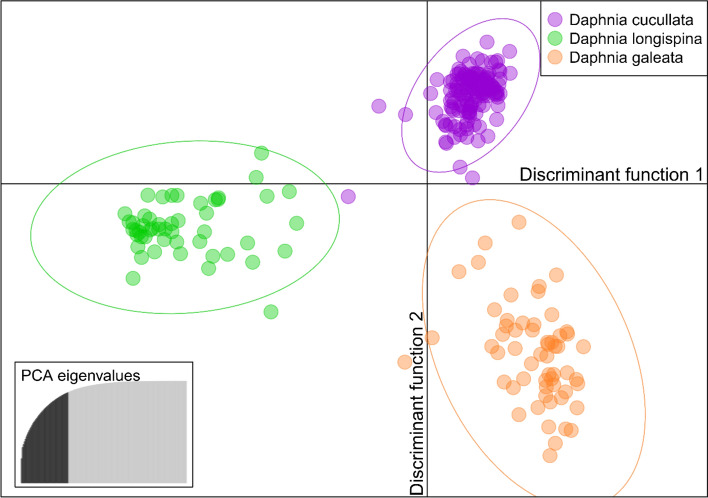
Results of Discriminant Analysis of Principal Components (DAPC), which clustered *Daphnia* genotypes inferred with microsatellite markers into three groups. *Daphnia* genotypes from contemporary samples from heated and control lakes and resting eggs from the heated lake MI were analysed together. Each dot represents one individual; inertia ellipses around groups are also presented. The inset plot presents PCA eigenvalues; those retained in the dimensional reduction step are indicated with black.

Based on analyses in DAPC and in STRUCTURE of the support for different numbers of clusters (K), we chose K = 3 as the optimal number of clusters within populations of contemporary *Daphnia* and resting eggs, both when they were analysed separately as well as in a combined analysis (Fig. 1, Supplementary Fig. S3 online). Through the inclusion of reference clones in the analyses, we were able to confirm that clustering based on K = 3 divided individuals into clusters that represented three distinct species: *D. cucullata*, *D. longispina*, and *D. galeata* (Fig. 2). Species assignment with DAPC and STRUCTURE was nearly identical, with only a few individuals indicated as hybrids (or backcrosses) by STRUCTURE and as pure species by DAPC.

**Figure 3:**
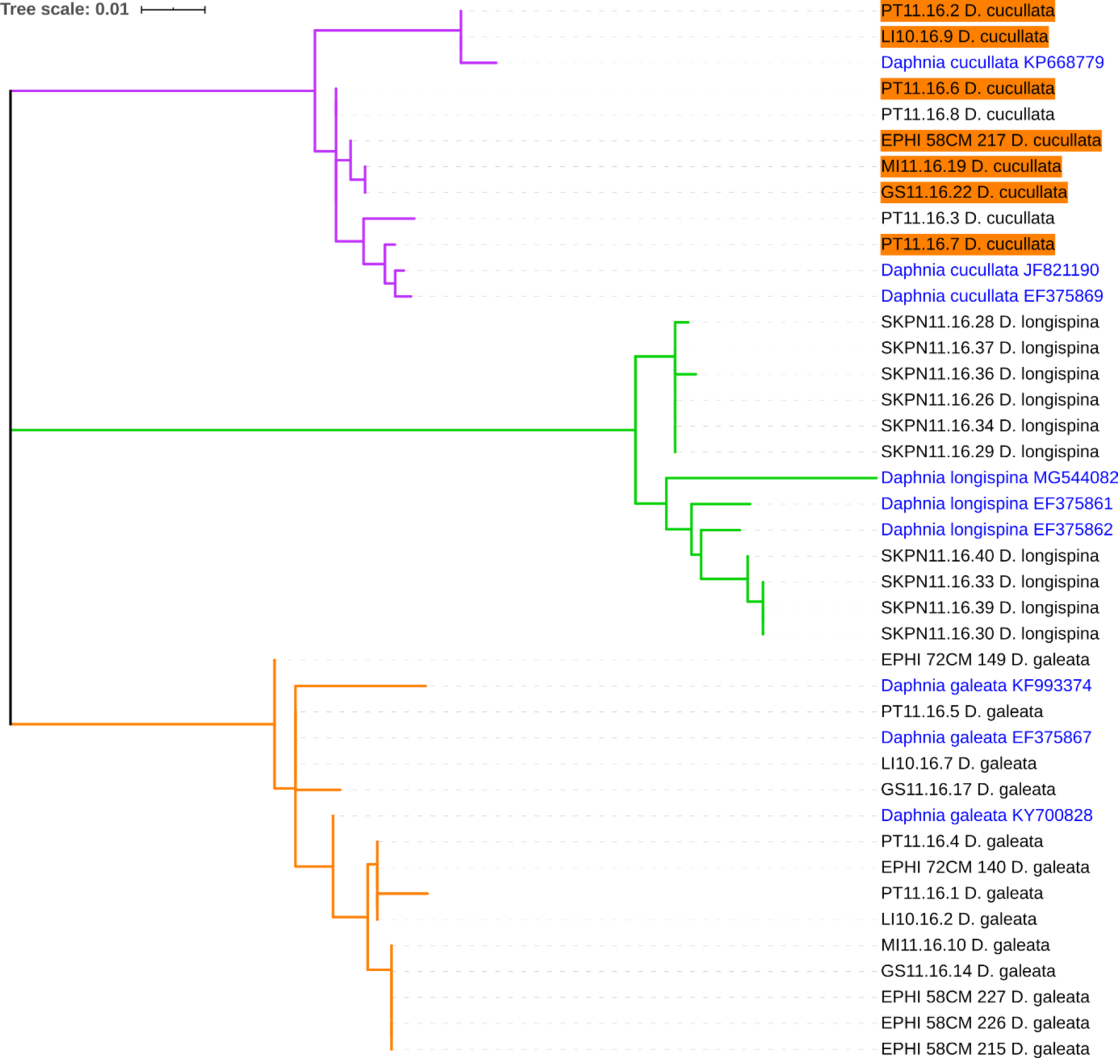
Maximum-likelihood phylogenetic tree computed with 1,000 bootstrap replicates, inferred from the alignment of partial *COI* gene sequences of 32 samples from the investigated lakes (black font) and 9 reference sequences downloaded from GenBank (blue font). Node colours indicate species assignment (inferred by reference sequences) and correspond to assignment by microsatellite data (Fig. 2). Orange boxes indicate samples assigned to *D. galeata* with microsatellites, but that clustered with *D. cucullata* by *COI*.

Using partial *COI* gene sequences for 32 samples obtained from the investigated lakes, we computed a phylogenetic tree that corroborated the presence of the three species (*D. cucullata*, *D. longispina*, and *D. galeata*) (Fig. 3). When we compared species assignment by *COI* with that from microsatellite data, we found a perfect match in the *D. galeata* and *D. longispina* clades, but mismatches in the *D. cucullata* clade. In this clade, 7 of 9 individuals assigned by barcoding to *D. cucullata* instead clustered with 100% accuracy to *D. galeata* in DAPC, and with 75–100% accuracy to *D. galeata* in STRUCTURE (Fig. 2, orange background). Because these discrepancies arose between species assignments based on mitochondrial (*COI*) sequences and those based on nuclear (microsatellite) DNA, it is likely that these individuals were hybrids.

**Figure 4:**
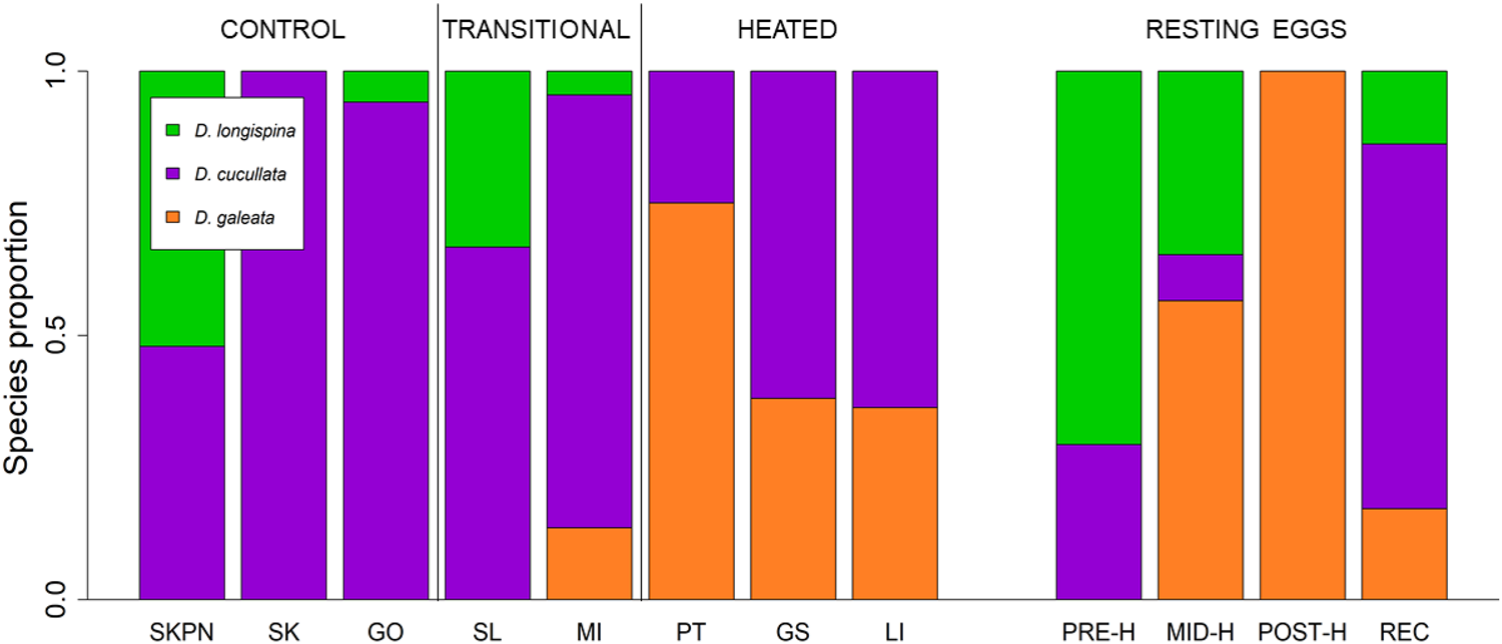
Species composition in the investigated lakes of contemporary samples and resting eggs of *Daphnia*, based on DAPC analyses. Contemporary samples are grouped as follows: three control lakes (SKPN, SK, GO), two transitional heated lakes (SL, MI), and three heated lakes (PT, GS, LI). Resting eggs are grouped as follows: PRE-H—produced before the onset of heating, MID-H—produced after the launch of the first plant but before the launch of the second power plant, POST-H—produced in the ca. 15 years following the launch of the second power plant, REC—produced recently, i.e. within 15 years of core collection.

According to both analyses of microsatellite data (STRUCTURE and DAPC), *D. longispina* was almost exclusively found in control lakes, while *D. galeata* was mostly confined to the heated lakes (with the least-heated lake, SL, conforming to the pattern of control lakes and the heated lake MI showing a transitional character, i.e. containing all three species; Fig. 4). *D. cucculata* was the dominant species in both types of lakes, although its share of the population varied among lakes (it ranged from 25% of individuals in PT to 100% in SK).

**Figure 5:**
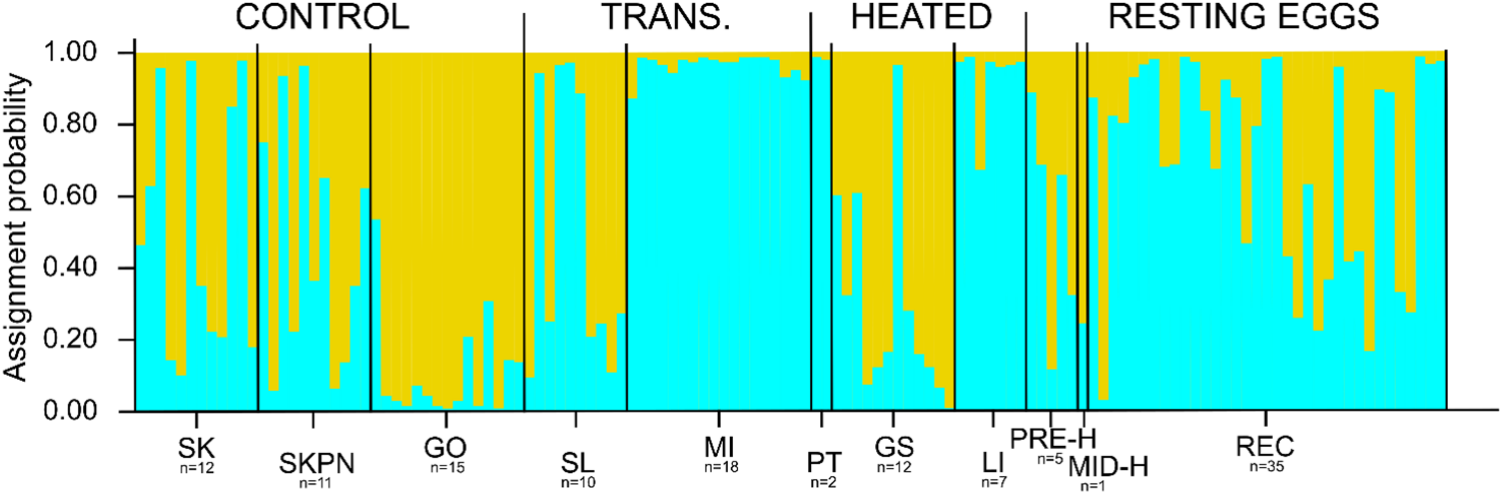
Plot of assignment probability of contemporary samples and resting eggs of *Daphnia cucullata* to two genetically distinct clusters (different colours), inferred with STRUCTURE. Each vertical line represents the assignment probability of one individual to two inferred clusters (light blue or yellow). Contemporary samples are grouped as follows: three control lakes (SK, SKPN, GO), two transitional (TRANS.) heated lakes (SL, MI), and three heated lakes (PT, GS, LI). Resting eggs are grouped as follows: PRE-H—produced before the onset of heating, MID-H—produced after the launch of the first plant but before the launch of the second power plant, REC—produced recently, i.e. within 15 years of core collection. There is no POST-H group, as it did not contain any individuals of *D. cucullata*. The n-value indicates sample size for each lake.

Within-species STRUCTURE analyses found that the optimal number of clusters for both *D. galeata* and *D. longispina* was K = 1, indicating little genetic structure within each of these two species and suggesting the existence of gene flow across their range in the sampled lakes (we do not provide graphical support, as a figure depicting K = 1 would not be meaningful). Instead, the optimal number of clusters for *D. cucculata* was two. A linear model with temperature as the explanatory variable (3 levels: control, transitional, and heated) and population as a random factor was used to test if individuals of *D. cucculata* differed in their estimated membership in the two clusters based on the temperature of their lake of origin; no significant difference was found (χ^2^ = 0.84, p = 0.66; Fig. 5). Pairwise F_ST_ values for contemporary *D. cucculata* samples ranged from 0.00 to 0.33 (Table 1). No pattern of isolation by distance within *D. cucculata* was detected (*r* = 0.22, *p* = 0.14).

**Table 1:** Pairwise F_ST_ values for all pairs of contemporary populations based on individuals of *D. cucculata*; ENA correction applied; significance level = 0.05; all values significant.

	SK	SKPN	GO	SL	MI	PT	GS
SKPN	0.059782						
GO	0.094996	0.072247					
SL	0.058211	0.076367	0.099724				
MI	0.144589	0.15588	0.200623	0.186023			
PT	0.03971	0.045013	0.135973	0.071922	0.033192		
GS	0.222188	0.162729	0.080433	0.208718	0.220905	0.334471	
LI	0.089108	0.078169	0.176584	0.157991	0.163908	− 0.06512	0.326313

Samples are ordered as follows: three control lakes (SK, SKPN, GO), two transitional heated lakes (SL, MI) and three heated lakes (PT, GS, LI).

### Resting eggs

**Figure 6:**
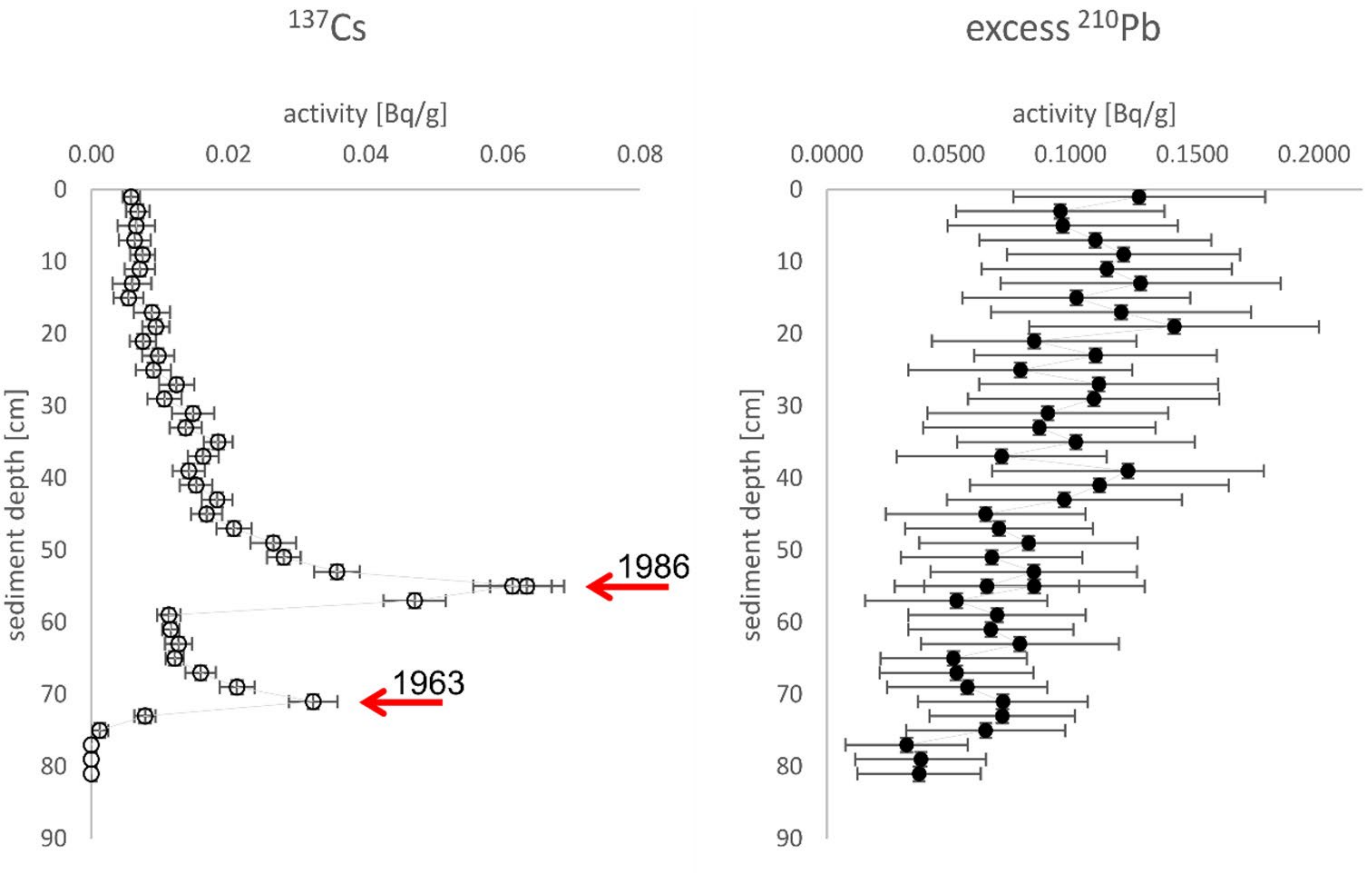
Downcore radioactivity of ^137^Cs (left panel) and excess ^210^Pb (right panel) in the sediment core from Lake Mikorzyńskie (MI). The prominent peaks in ^137^Cs are assigned to 1986 (upper) and 1963 (lower). The average rate of sediment accumulation was calculated to be between < 0.7 cm/year for deeper sediments to > 1.1 cm/year for more recent sediments. The vertical error bars refer to analysed sediment sample thickness, while the horizontal bars depict 2-sigma uncertainty.

Prior to investigating resting eggs, we analysed the sediments and computed an age-depth model. The sediment core from heated Lake Mikorzyńskie (MI) was composed of carbonate-rich gyttja, with the sediments laminated in the upper part. Analysis of the downcore radioactivity of ^137^Cs (Fig. 6) revealed prominent peaks that were assigned to the years 1986 (upper) and 1963 (lower); in the deepest samples, ^137^Cs was not detected (pre-1952). These time-markers were used to create an age model and calculate the average rate of sediment accumulation, which increased with time from < 0.7 cm/year in the 1950s to > 1.1 cm/year after 1986. These sediment accumulation rates and their change over time were also supported by analyses of excess ^210^Pb (Fig. 6) and of annual lamination (with an average lamina thickness of ca. 1 cm). The age model suggested that the sediments that formed prior to lake heating were found below a depth of 72 cm. Thus, the increase in the sediment accumulation rate can be linked to that period. The age-depth model for sediments in Lake Skulska Wieś was adapted from work by Woszczyk et al.^31^.

Using microsatellite data, we investigated the community composition of the resting eggs produced by *Daphnia* in MI before and after the launch of two power plants, to test if the heating resulted in any shifts in the community structure of the lake. Temporal changes in community structure conformed with the patterns we observed in the contemporary samples: prior to heating, MI was inhabited by *D. cucullata* and *D. longispina*, whereas after the onset of heating *D. galeata* appeared, entirely dominating the community after the launch of the second power plant (Fig. 4). In recent years, it appeared that the proportion of *D. galeata* in the ephippial community of MI had decreased, and the lake was recolonised by *D. longispina* and *D. cucullata*.

**Figure 7:**
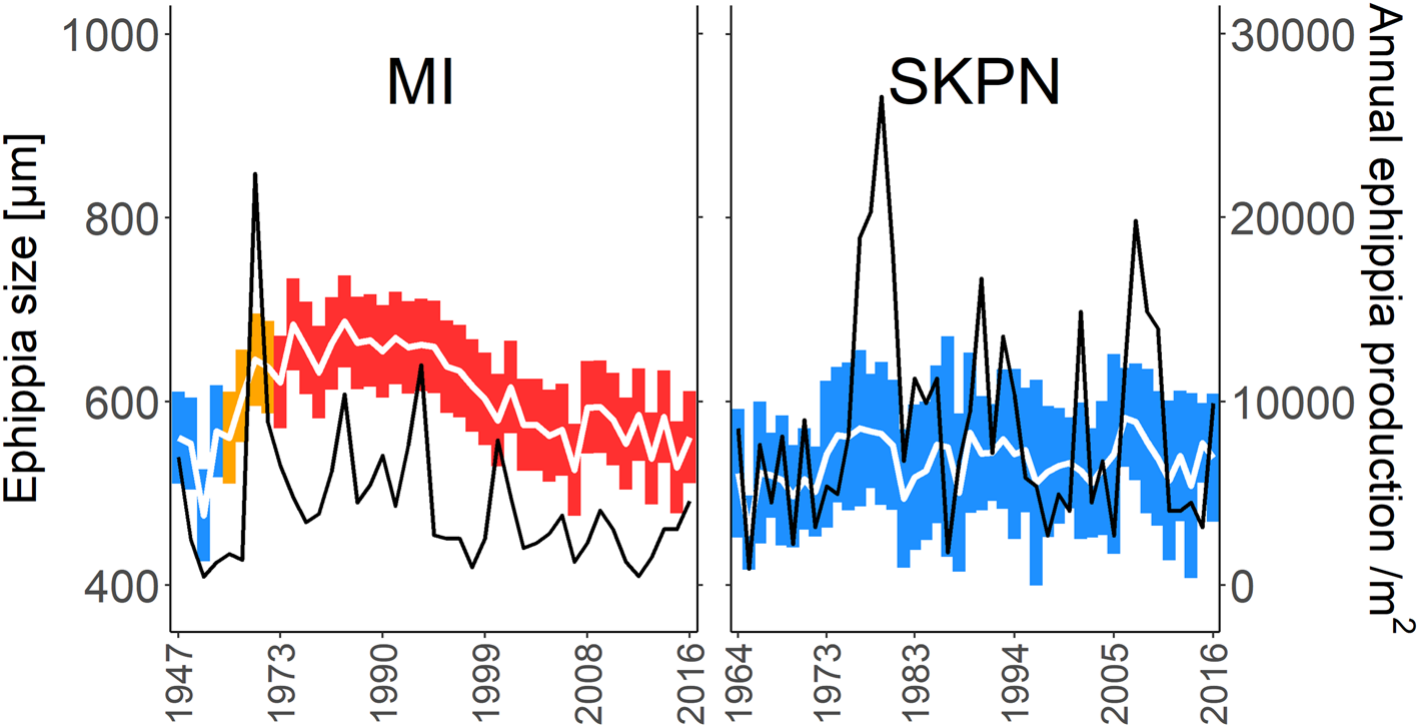
Size (white line and bars indicate mean ± SD, respectively) and annual flux of ephippia per m^2^ of sediments (black line) of *Daphnia* individuals extracted from sediments produced in the analysed time points (presented on x-axis) in heated Lake Mikorzyńskie (MI) and control Lake Skulska Wieś (SKPN). Colours of bars indicate the thermal status of the lake at a given timepoint (blue—non-heated, yellow—heated (after the launch of the first power plant), red—heated (after the launch of the second power plant). Age model is based on the present study for MI and Woszczyk et al.^31^ for SKPN.

Overall, the size and abundance of ephippia from the *Daphnia* community dramatically increased in MI after the onset of heating, but then both parameters gradually decreased (size—year correlation: *r* = − 0.81, *t* = − 7.79, *p* < 0.001; abundance—year correlation: *r* = − 0.51, *t* = − 3.33, *p* = 0.002). Instead, in the control lake SKPN, both the size and abundance of ephippia were stable across the entire analysed time span (size—year correlation: *t* = 1.68, *p* = 0.099; abundance—year correlation: *t* = 0.27, *p* = 0.786, Fig. 7).

## Discussion

In line with our expectations, we found that *D. galeata* prevailed in the heated lakes (where it co-occurred with *D. cucullata*), while the control lakes were inhabited by *D. longispina* (also co-occurring with *D. cucullata*). In *D. cucullata*, the lack of evidence for isolation by distance or for temperature-related genetic structure suggests the existence of gene flow between populations in heated and control lakes. This gene flow could be facilitated by the short distances and existing waterways between the heated and control lakes, which enable the transfer of *Daphnia* or their resting eggs via watercourses and numerous other vectors^32^. For this reason, it would seem likely that the separation of *D. galeata* and *D. longispina* is maintained by temperature-mediated selection rather than by a limited possibility for migration. Genetic data from subfossil resting eggs confirm this conclusion: before MI was heated, its *Daphnia* community was composed of *D. longispina* and *D. cucullata*, resembling the contemporary communities of control lakes. Instead, after heating was initiated, *D. galeata* dominated the lake. In the more-recent sediments of MI, the taxonomic structure of the resting egg community was similar to the contemporary community of active daphnids, which supports the correspondence between subfossil records and the active *Daphnia* community. The high degree of consistency between the community restructuring patterns observed in contemporary samples and those found in resting eggs gives us confidence in proposing that the observed alterations in *Daphnia* community composition were induced by the temperature increase.

Based on analyses of microsatellite markers, patterns of *Daphnia* community structure appear to reflect the division of the studied lakes into three groups based on temperature regime: the warmest lakes, heated throughout the entire water column (PT, LI, GS); transitional lakes, which are deep and have heated surfaces (SL and MI); and the non-heated control lakes (GO, SK, SKPN; Fig. 4, Supplementary Fig. S1). One of the transitional lakes, SL, is heated only periodically and rather weakly, which might explain why the *Daphnia* community in this lake was more like those of the control lakes. In turn, MI is strongly heated, but its large depth and stratification might provide thermal refuge for sensitive organisms. This could be why its overall taxonomic composition was similar to heated lakes, but with a small contribution from *D. longispina*, a species specific to control lakes. It should also be noted that MI was the only lake that harboured all three *Daphnia* species. Its transitional character, between that of the shallow heated lakes and that of the control lakes, possibly creates favourable conditions for the establishment of all three species. It seems plausible that deep, stratified lakes could serve as refuges for species that are less adapted to warm conditions (like *D. longispina*) under climate warming^33^.

Our finding that *D. galeata* and *D. longispina* were restricted to warmer and colder environments, respectively, is consistent with that of Keller et al.^27^, who showed that these two species are also segregated among alpine lakes. *D. galeata* is found in warmer lakes of the southern Alps, and those authors specifically identified temperature as the factor that explained the dominance of *D. galeata* in those habitats. In China, *D. galeata* is present at low altitudes, and absent from intermediate- or high-altitude lakes, although hybrids of this species can survive in higher-altitude (and thus colder) lakes^34^. Additionally, it has been reported that warm, iceless winters promote the population growth of *D. galeata* and *D. galeata* × *D. longispina* hybrids over that of pure *D. longispina*, whereas after harsh winters *D. longispina* gains an advantage^29^. In line with these reports, our results also suggest that *D. galeata* may be able to displace *D. longispina* under conditions of climate warming. By genotyping the resting eggs that had been produced before lake heating, we found that *D. galeata* was not present in the heated lake MI prior to disturbance of the thermal regime. Therefore, we conclude that, facilitated by an increase in temperature, lakes inhabited by *D. longispina* may be successfully invaded by *D. galeata*, and also that *D. galeata* has a competitive advantage over *D. longispina* under warmer conditions. This in turn underscores the importance of the ‘change of space’ mechanism rapidly following climate change^14,19,21^. The spread of species and genotypes from a warmer climate seems to be facilitated not only by the increased temperature of local habitats, but also by the poor biotic resistance of local communities, which is limited by disturbances in community structure (e.g.,^35^) and by the poorer performance of indigenous populations compared to that of invaders (e.g.,^23,25,26^).

Our investigation of the pool of resting eggs yielded results similar to those of Zeis et al.^29^ both in terms of changes in species composition as well as a shift in overwintering strategy. The size and number of ephippia in MI increased rapidly immediately after the onset of heating, but both then decreased over time (Fig. 7). An increase in the size and number of ephippia in a lake is a hallmark of the arrival of *D. galeata*, as *D. galeata* from the heated lakes are larger than *D. cucullata* and *D. longispina* (personal observation). Increased ephippia production after the start of heating can be additionally explained by enhanced productivity in the warmer lake^36,37^. Later, a gradual reduction in the size and number of ephippia could indicate a decline in the contribution of the larger *D. galeata* to the bank of resting eggs (observed also by Tsugeki et al.^37^). In line with this hypothesis, we found a shift in relative species abundance in the pool of resting eggs, from the domination of *D. galeata* directly after the onset of heating (POST-H) to an increased prevalence of the smallest species, *D. cucullata*, in recently produced ephippia (REC). Most likely, this represents a change in the overwintering strategy of *D. galeata* from production of sexual resting eggs to parthenogenesis. A shift to active overwintering seems to explain the data, and a similar pattern has previously been reported for *D. galeata* in response to warm, iceless winters^29,37^. Maintaining parthenogenesis over mild winters provides *D. galeata* with an advantage over competitors in spring^29^. Another potential explanation for the reduced production of ephippia could be an increased number of hybrids, which produce fewer resting eggs^38^. However, if hybrids were abundant in these populations, they likely would have appeared as additional groups in the microsatellite analyses, which was not the case here. Alternatively, they might have demonstrated species assignment to two clusters, representing their parental species, but this was the case for just a few individuals. As such, we expect that hybrids might contribute to the phenomenon of reduced ephippia production, but it does not appear that they play a major role.

We compared populations of *D. cucullata* from the heated and control lakes to detect within-species genetic structure (the ‘change of self’ hypothesis), but found no pattern of variation that corresponded with changes in thermal regime. This result may indicate a lack of warming-mediated selection acting on *D. cucullata* populations in the heated lakes. It is reasonable to assume that the thermal selection pressure on *D. cucullata* is lower than on other species, as *D. cucullata* is the smallest species of the *D. longispina* complex^39^ and smaller individuals are seemingly less affected by stress caused by temperature increase^40,41^. It is thus possible that this species might be more resistant to the applied warming of ca. 3–4 °C. However, sediment records indicated that *D. cucullata* disappeared from MI immediately after the onset of heating, and only returned years later, which would suggest that this species was in some way affected by the warming. One possible explanation is that it never really disappeared, but that, under strong ecological pressure (temperature increase or new competitor—*D. galeata*), its abundance dropped below the level of detection and remained low until the population adapted. However, such strong selective pressure would almost certainly have had an influence on the population structure of *D. cucullata*, and since we did not observe clear divergence between the populations in heated and control lakes, we consider this scenario to be unlikely. Another possibility is that *D. cucullata* did truly disappear from MI immediately after the onset of heating as a result of being smaller, and therefore a worse competitor, than the invader *D. galeata* (an example of size-efficiency theory^42^). Subsequently, its gradual recolonisation might have been facilitated by increasing predation pressure from fish on the larger *D. galeata*, which was enhanced by the temperature increase^43–47^. Selective culling of *D. galeata* may have allowed for the successful dispersal of *D. cucullata* from the control lakes, which would explain the genetic similarity between populations in the heated and control lakes. *D. cucullata* seems to thrive in the heated lakes during summer^48,49^, when fish predation, and therefore size reduction in *Daphnia,* is the strongest^50^. The coexistence of these two species in the heated lakes could be facilitated by phenological cycling in abundance, driven by intense predation on *D. galeata* in summer and the competitive inferiority of *D. cucullata* in winter.

The structure of zooplankton communities is frequently shaped by size-selective predation^42^. In the case of *D. galeata* and *D. longispina*—species with overlapping ranges of body size—it is unlikely that their segregation between the heated and control lakes is maintained by predation. Moreover, communities of *Daphnia* reach similar body sizes in both heated and control lakes, indicating a similar degree of size-selective predation pressure among these lakes (personal observation). Additionally, fish communities are similar in the heated and transitional lakes^51^, and fish frequently migrate among them^45^ (no data on fish stocks in control lakes are available). Therefore it seems unlikely that size-selective predation is the main factor behind the differences between *Daphnia* communities in transitional and heated lakes. Besides temperature, the distributions of *D. galeata* and *D. longispina* might also be shaped by trophy^27^ and food quality^52^. However, all of the lakes investigated here are eutrophic, and there does not seem to be a gradient in trophic state between the heated and control lakes (see TSI(SD) in Supplementary Table S1 online). Regarding food quality, there is evidence that *Daphnia* from heated lakes can more effectively cope with filamentous cyanobacteria, which are generally considered a poor quality and difficult-to-process food source^53^. This could be advantageous if cyanobacterial blooms intensify under global warming conditions^2^, but so far such blooms have not been frequently observed in heated lakes^54^. Furthermore, subfossil data confirmed that if any environmental parameters other than temperature played a role in the shift in *Daphnia* community structure, they co-occured in time with, and were most likely related to, lake heating. It is probable that the temperature increase affected not only zooplankton, but the entire ecosystem, including the quality and quantity of algae, fish, and other organisms as well^6,33^. Therefore, we argue that the observed patterns are a result of both direct and indirect effects of temperature increase, which should be considered jointly in climate change projections.

Temperature-mediated selection can result in an adaptive response in a population in a relatively short time span^30,55,56^. However, our results indicate that such selective pressure also induces changes in the community structure, in this case, favouring *D. galeata* over *D. longispina*. In addition, *Daphnia* from heated lakes were found to cope better than those from control lakes with the presence of filamentous cyanobacteria^53^. This ability can contribute to the competitive superiority of *D. galeata* over *D. longispina* under conditions of global warming. The competitive exclusion of some *Daphnia* species in increased temperatures might, however, be averted through hybridisation, which could preserve their gene pool for a period of time in lakes undergoing warming. Hybridisation could also produce novel genetic variants to enhance the resistance of the entire community^57,58^, and especially of *D. longispina*, via introgression of genes that improve resistance to elevated temperature. However, this buffering effect might be limited because, as shown here and by Zeis et al.^29^, an increase in temperature reduces the overall frequency of sexual reproduction, and hence hybridisation as well. A lack of genetic recombination due to constrained sexual reproduction may reduce the general ability of *Daphnia* to adapt to the changing environment.

To summarise, by taking advantage of a system of lakes that have been heated for decades by about 3–4 °C—an amount corresponding to most models of climate change^1^—we are able to describe an approximate scenario for the potential consequences of climate change in *Daphnia* communities that inhabit temperate lakes. We observed a rapid invasion by *D. galeata*, facilitated most likely by temperature increase. We also detected a time-lagged response through the trophic cascade, enabling recolonisation of the species that disappeared immediately after the temperature increase. This pattern points to two major conclusions: i) existing biotic resistance might be insufficient to counteract an invasion under climate change conditions, and ii) immediate and long-term outcomes of environmental disturbance can vary strongly, and therefore, long-term patterns extrapolated from short-term observations should be treated cautiously^59^. Events such as an ecosystem-wide increase in temperature affect each element of a trophic cascade, and the full complexity of trophic interactions must be taken into account in order to reveal the fate of a focal population. Restoration of a trophic cascade after such a disturbance is likely a long-term process; for this reason, observations of ecosystems that are undergoing long-term warming are particularly valuable for increasing the accuracy of predictions. Although we cannot provide a detailed mechanism of the ecosystem response to temperature increase in this study, we report the long-term outcome for the focal community of all the processes that occurred due to temperature increase.

Our results suggest that progressive warming will alter the structure of zooplankton communities, including their overwintering strategies and frequency of sexual reproduction. Temperature increase can promote the invasion of alien species or genotypes, which occurs more rapidly than the adaptation of local communities. In the worst-case scenario, we might expect that global warming will lead to the competitive exclusion of species that fail to adapt quickly enough, significantly affecting the diversity of local communities. Furthermore, the abandonment of sexual reproduction could increase the vulnerability of *Daphnia* (and zooplankton in general) to environmental stress, threatening the future of populations and communities. Because *Daphnia* plays a vital role in freshwater ecosystems^60^, its fate will have a bearing on the functioning of entire ecosystems. Planktonic organisms that inhabit stratified lakes with deep-water refuges might be less susceptible to the negative consequences of temperature increase^61^, but in the case of shallow polymictic lakes, temperature increase is likely to have far-reaching consequences.

## Methods

### Study area

The heated lakes used in this study are situated in western Poland, near the city of Konin, in close proximity to lignite (brown coal) open cast mines. In this region, two lignite-combusting power plants (Pątnów, operating since 1958, and Konin, since 1970) use the water of five nearby lakes as coolant. This makes the shallow lakes Licheńskie (LI), Gosławskie (GS), and Pątnowskie (PT) (see Supplementary Fig. S1 and S3 online, red) warmer than natural, non-heated lakes by ca. 3–4 °C, throughout the entire water column. Two deeper lakes, Wąsowsko-Mikorzyńskie (MI) and Ślesińskie (SL), are heated by power plants (at the same rate) only in the epilimnion (Supplementary Fig. S1 and S3 yellow). In the latter case, heating occurs only during the warmest months of the year, when the power plants’ cooling systems require an increased amount of water. All five heated lakes are interconnected by water channels, which allows, to some extent, for the migration of organisms among lakes in the system (Supplementary Fig. S2). Some basic information (e.g., surface area, depth, temperature in fall, location coordinates) about the investigated lakes is presented in Supplementary Table S1 online. Details concerning the system of heated lakes are described elsewhere (e.g.,^54,62^). Within this same region, there are also several lakes that are not affected by heating. Three of these were included in this study as control lakes, namely, Goplo (GO), Skulskie (SK), and Skulska Wieś (SKPN). These lakes lie in close proximity to the heated lakes (maximum distance between a control and a heated lake is ca. 22 km, minimum distance is ca. 6.5 km). The control lakes are connected with the heated lakes through water channels, either permanently (GO-SL) or periodically (SK-SL). Due to the specific spatial arrangement of these lakes (shallow heated lakes—MI–SL—control lakes, Supplementary Fig. S2) and slightly different thermal regimes of MI and SL (both have deep water thermal refugia, and SL is only periodically heated), these latter two lakes have a transitional character, between the shallow, heated lakes and control lakes. All the examined lakes are eutrophic, subjected to the same microclimate, and have similar catchment use; we therefore believe that difference in water temperature is the strongest driver of variation in the structure and functioning of biota among these groups of lakes.

### Daphnia sampling

Five heated lakes and three non-heated control lakes were sampled in autumn (October or November) of 2016. *Daphnia* samples were collected by vertical hauling of a 100-µm plankton net throughout the water column in the pelagic zone of each lake (sampling sites are indicated in Supplementary Fig. S2). Samples were preserved with 96% ethanol to a final concentration of ca. 80% ethanol. Samples were then examined under a dissecting microscope and 24 *Daphnia* from each lake (25 in case of SKPN) were randomly chosen. When possible, these individuals were identified to the species level following the diagnostic features described in the species identification key^63^. Each individual was then drawn by tail spine or antenna into a clean tube filled with digesting buffer using flame-sterilised forceps.

### Ephippia sampling

Two sediment cores were collected from transitional, heated Lake Mikorzyńskie and control Lake Skulska Wieś (coring locations are indicated in Supplementary Fig. S2). A 1-m-long core from Lake Mikorzyńskie and 0.5-m-long core from Lake Skulska Wieś, both with an internal tube diameter of 90 mm, were recovered using a core sampler (Uwitec, Mondsee, Austria). Prior to processing, the sediment cores were stored in darkness at 4 °C. Then, the cores were cut lengthwise into two halves, described, sliced into 2-cm-thick slices, and packed into plastic zip bags. The samples from one half were used for ^137^Cs- and ^210^Pb-based sediment dating (only in the case of MI), while the other half was used for the characterisation of resting eggs (ephippia).

Sediment dating of the core from Lake Mikorzyńskie was performed using well-established methods involving the use of ^137^Cs and ^210^Pb radioisotopes (see e.g.,^64,65^). ^137^Cs is of anthropogenic origin and was first introduced into the environment in measurable amounts in ca. 1952. Maximum activity usually corresponds to 1963, related to fallout from numerous nuclear bomb tests prior to the surface Test Ban Treaty, and 1986, related to the Chernobyl event. ^210^Pb is instead a natural radionuclide, which arrives in lake sediments partly from atmospheric fallout (called excess ^210^Pb). By taking into consideration its rate of decay (t_1/2_ = 22.3 years), it is possible to use this isotope to assess sediment accumulation rates during the last ca. 100–150 years. The sediment samples were dried and ground, and levels of ^137^Cs, ^210^Pb, and excess ^210^Pb (calculated as the difference between measured total ^210^Pb and the average of measured ^214^Pb and ^214^Bi) were quantified using the gamma spectrometer Canberra GX2520^66^. For age model calculation, we used the models available in the *serac* package^65^. The age-depth model for Lake Skulska Wieś was adapted from Woszczyk et al.^31^.

For ephippia isolation, core slices were rinsed on a 150-µm sieve to wash out mineral and small organic sediments. The remaining material was flushed on a petri dish. Ephippia were counted and measured under a dissecting microscope, and then transferred with flame-sterilised forceps into tubes filled with 96% ethanol. The flow-through from the first five sieved samples was examined to determine if any ephippia went through the sieve, but none were detected. For molecular analyses, a total of 254 ephippia from MI were divided into four groups: those produced before lake heating (PRE-H), after the launch of the first power plant (MID-H), up to 15 years after the launch of the second power plant (POST-H), and recently (up to 15 years before coring—REC). Ephippia were transferred onto a petri dish under a dissecting microscope, each in a separate droplet of distilled water, and opened with flame-sterilised forceps and entomological needle. If the opened ephippium contained two eggs, one was transferred to a new droplet with a micropipette to avoid contamination. If neither of the eggs was damaged, each was crushed in a separate water droplet with a pipette tip and transferred with a micropipette into a separate tube that contained digesting buffer. If one of the eggs was damaged before or during ephippium opening, only the damaged one was transferred for DNA isolation; the intact egg was discarded due to the possibility of contamination from the damaged egg. If an ephippium contained only one egg, it was taken for DNA isolation regardless of if it was damaged or not. There were no ephippia in which both eggs were mechanically damaged.

We analysed the trend in average size (measured as dorsal length) and number of ephippia of *Daphnia* communities (with no division into separate species) after the onset of warm water discharge from power plants into MI, and for all available parallel time points from the sediment core of SKPN. Trends in size and number of ephippia were analysed separately for each lake with Pearson’s product-moment correlation. In the case of ephippia abundance, data from both lakes required prior log transformation to obtain a normal distribution of residuals.

### Molecular methods

DNA was extracted using spin columns and the DNeasy Blood and Tissue Kit (Qiagen GmbH, Hilden, Germany) according to the manufacturer’s protocol, then eluted with 100 µL of 10 mM Tris, pH 8.0.

### Mitochondrial DNA

To assign individuals to species, we amplified a partial sequence of the mitochondrial cytochrome oxidase subunit I (*COI*) gene, which is species-specific in *Daphnia*^28^, from a subset of current-day populations (16 and 10 individuals from the heated and control lakes, respectively) and the resting eggs from sediment cores (6 eggs). The following primers were used: bcdF01: 5′-CATTTTCHACTAAYCATAARGATATTGG-3′ and bcdR04: 5′-TATAAACYTCDGGATGNCCAAAAAA-3′^67^. *COI* gene sequences were amplified from 1 μL DNA template in 5 μL of PCR reaction mix, containing 2.5 μL Type-it Microsatellite Kit (Qiagen GmbH, Hilden, Germany), 0.25 μmol/L of each primer, and 2 μL of water. Thermocycling parameters were: one cycle of 5 min at 95 °C; 35 steps of 30 s at 95 °C, 1 min at 50 °C, and 1 min at 72 °C; and a final step of 5 min at 72 °C. The PCR product was visualised by electrophoresis on 1% agarose gel, purified using Exonuclease I and Alkaline Phosphatase (Thermo Scientific, San Jose, USA) for 15 min at 37 °C and 15 min at 80 °C, and Sanger sequenced using the BigDye Terminator v3.1 kit and an ABI Prism 3130XL Analyzer (Applied Biosystems, Foster City, CA, USA).

### Microsatellites

All individuals were screened for variation at 20 microsatellite loci that had been previously used for identification of the *Daphnia longispina* complex^68^. Loci were divided into five groups, enabling the amplification of products in five multiplex PCR reactions (Multiplex 1: SwiD4, SwiD10, SwiD16; Multiplex 2: SwiD1, Dp512, Dp196NB, Dgm109; Multiplex 3: Dp519, SwiD8, SwiD12, Dp281NB; Multiplex 4: SwiD2, SwiD5, SwiD15, DaB10/15; Multiplex 5: DaB10/14, Dgm101, Dgm105, Dgm112, SwiD14); primer sequences can be found in Brede et al.^68^. PCR reactions were conducted in a 5 μL mix that contained 2.5 μL Type-it Microsatellite Kit mix (Qiagen GmbH, Hilden, Germany), 0.2 μmol/L of each primer, 1 μL of DNA template, and enough water to make 5 μL. Thermocycling parameters were: one cycle of 5 min at 95 °C, followed by 35 steps of 30 s at 95 °C, 1 min 30 s at 55 °C, and 30 s at 72 °C, with a final step of 30 min at 60 °C. PCR products were diluted with 70 μL of water, and electrophoresis was conducted with an ABI Prism 3130XL Analyzer. Allele scoring was conducted using STRand software^69^ v. 2.4.110 (https://www.vgl.ucdavis.edu/STRand) and integer alleles were binned using the R package MsatAllele^70^. Using these microsatellite markers, we successfully genotyped 133 individuals from contemporary samples and 111 ephippial eggs, which were further analysed with STRUCTURE and DAPC.

### Phylogenetic and population genetic analyses

The partial sequences of *COI* were manually checked and aligned using the MUSCLE algorithm^71^ implemented in MEGA X^72^. For reference, three additional *COI* sequences were downloaded from GenBank for each species of the *D. longispina* complex present in the studied lakes (accession numbers: KP668779, JF821190, EF375869, MG544082, EF375861, EF375862, KF993374, EF375867, and KY700828); these were used as species-specific references for alignment and tree construction. A maximum-likelihood tree was constructed in MEGA X, using the Tamura three-parameter method for the calculation of evolutionary distance. One-thousand bootstrap replicates were computed for tree construction.

Two types of analyses were performed on the microsatellite data. To determine the most probable number of genetically distinct clusters (K), the data were analysed with STRUCTURE 2.3.4^73^, using an admixture model, uncorrelated allele frequencies, and allowing for null alleles. The burn-in period and the number of Markov Chain Monte Carlo (MCMC) steps were both set to one million each. Ten runs were performed for each K-value, which ranged from one to twelve (twelve being the number of sampling events, differing in lake of origin, time, and/or mode of sampling). Additionally, we performed separate analyses on groups of individuals that were identified as *D. cucculata*, *D. galeata*, or *D. longispina* based on morphology and mitochondrial sequences (Fig. 2) in order to detect any possible structure within species. In the within-species analyses, only individuals with 100% membership in the cluster of a given species were considered. Thus, it should be kept in mind that in this way we very likely excluded hybrid individuals from these analyses. Structure Harvester^74^ was used to calculate ΔK, a measure that enables estimation of the most probable number of genetically distinct clusters based on STRUCTURE results^75^. ΔK is a statistic based on the second-order rate of change of the probability of data for different K values, the maximum of which indicates the optimal value of K.

Discriminant Analysis of Principal Components (DAPC^74^) was used to assess and visualise the relationships among distinct clusters. First, the optimal number of clusters was estimated with the K-means algorithm (K-value), based on a comparison of values of the Bayesian Information Criterion (BIC) among analyses performed for different values of K, ranging from one to twelve. When choosing the optimal number of clusters, we took into consideration both the size of ΔBIC and the lowest BIC value. The optimal K-value was the one with a low BIC value, but past which ΔBIC was very small and further increases in K provided only poor explanatory power. DAPC was performed in R, using the package *adegenet*^76^. An additional DAPC was conducted with the addition of reference clones (determined morphologically and genotyped at microsatellites, allozymes, and *COI*) that originated from the heated and control lakes studied here: three of *D. cucculata*, three of *D. galeata*, and three of *D. longispina.* We also included one reference clone of *D. galeata* (G100) and one clone of *D. longispina* (H7) that were obtained from Eawag, the Swiss Federal Institute of Aquatic Science and Technology in Switzerland. Reference clones were used to verify if individuals clustered by species and, if so, to determine the cluster that represented each species.

In contemporary samples, *D. cucculata* was the only species found in both the heated and control lakes. Using FreeNA^77^, we calculated pairwise F_ST_ values between populations from different lakes, adjusted for the presence of null alleles with the ‘excluding null alleles’ (ENA) correction^77^. A Mantel test, implemented in IBD v.1.5^78^, was used to test for a correlation between the genetic (linearised pairwise F_ST_) and linear log-geographic distance in contemporary populations from different lakes.

## Supplementary information

Supplementary information
