# Supplementary material for: Temperature increase altered Daphnia community structure in artificially heated lakes: a potential scenario for a warmer future
Source: Sci Rep. 2020 Aug 18;10:13956. doi: 10.1038/s41598-020-70294-6 (PMC7434883; doi:10.1038/s41598-020-70294-6)
Supplement: Supplementary file 1 — Supplementary information [file 41598_2020_70294_MOESM1_ESM.pdf]

Temperature increase altered *Daphnia* community structure in artificially heated lakes: a potential scenario for a warmer future

Authors: Marcin K. Dziuba[1,2]*, Magdalena Herdegen-Radwan[2], Estera Pluta[1], Łukasz Wejnerowski[1], Witold Szczuciński[3], Slawek Cerbin[1]

SUPPLEMENTARY MATERIAL

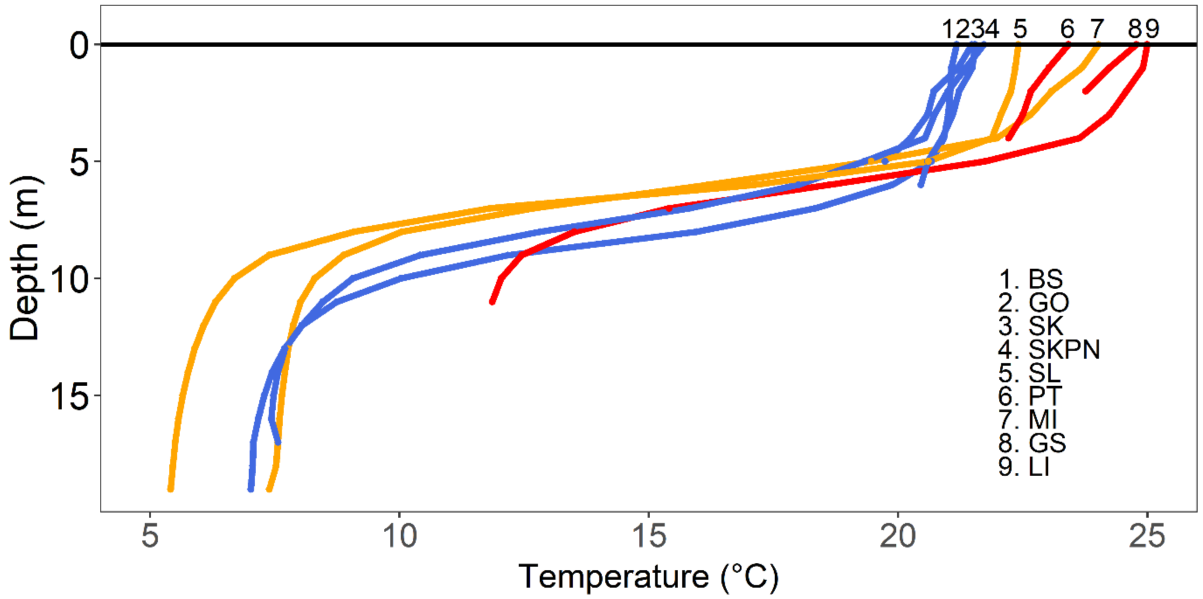

Fig. S1. Average summer temperature profiles of heated shallow (red) lakes Pątnowskie (PT), Gosławskie (GS) and Licheńskie (LI), heated deep (yellow) lakes Ślesińskie (SL) and Wąsowsko-Mikorzyńskie (MI) and control (blue) lakes Gopło (GO), Skulskie (SK), Skulska Wieś (SKPN) and Budzisławskie (BS; not used in this study).

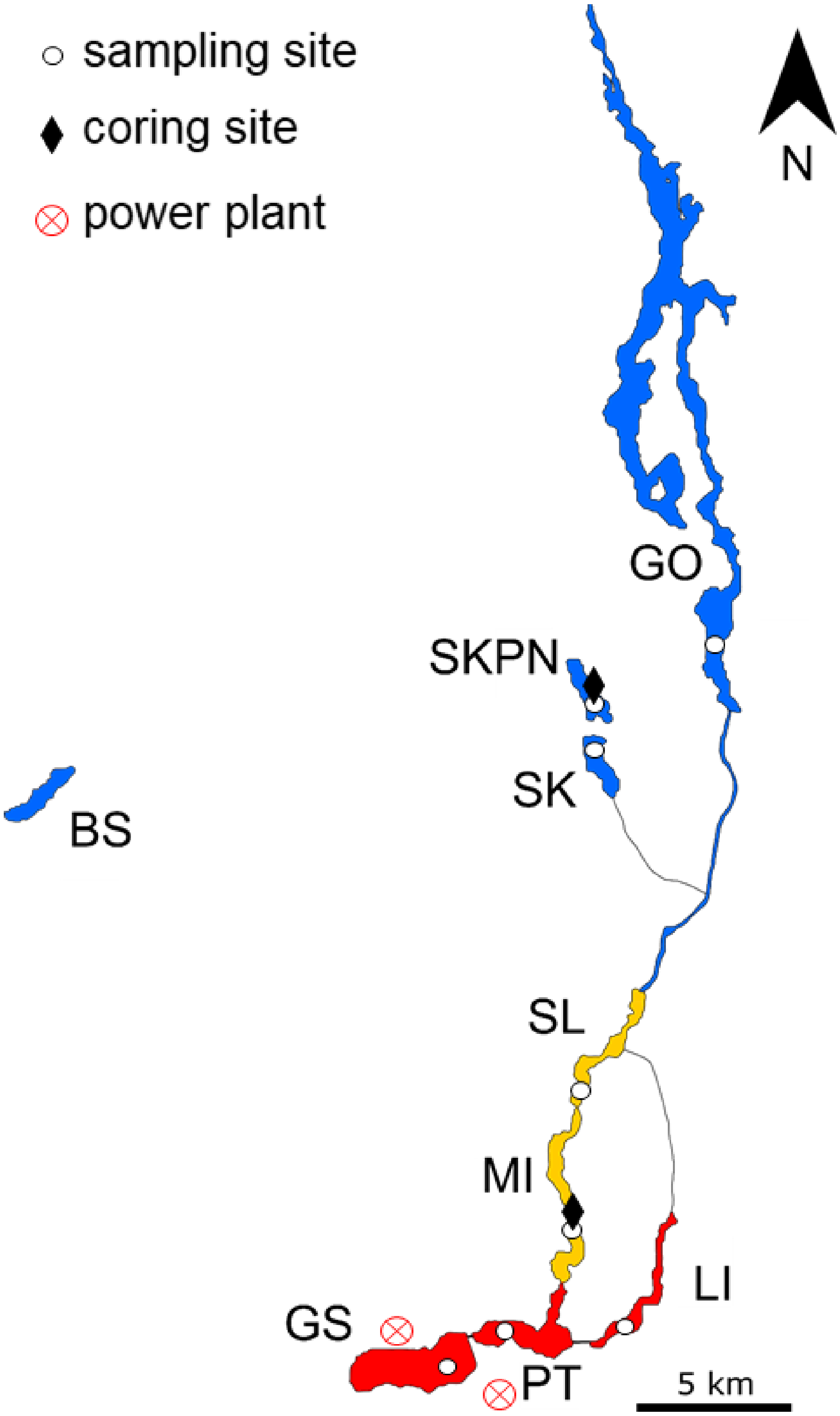

Fig. S2. Map of the system (geographical coordinates: 52.4° N; 18.3° E) of heated shallow (red) lakes Pątnowskie (PT), Gosławskie (GS) and Licheńskie (LI), heated deep (yellow) lakes Ślesińskie (SL) and Wąsowsko-Mikorzyńskie (MI) and control (blue) lakes Gopło (GO), Skulskie (SK), Skulska Wieś (SKPN) and Budzisławskie (BS; not used in this study).

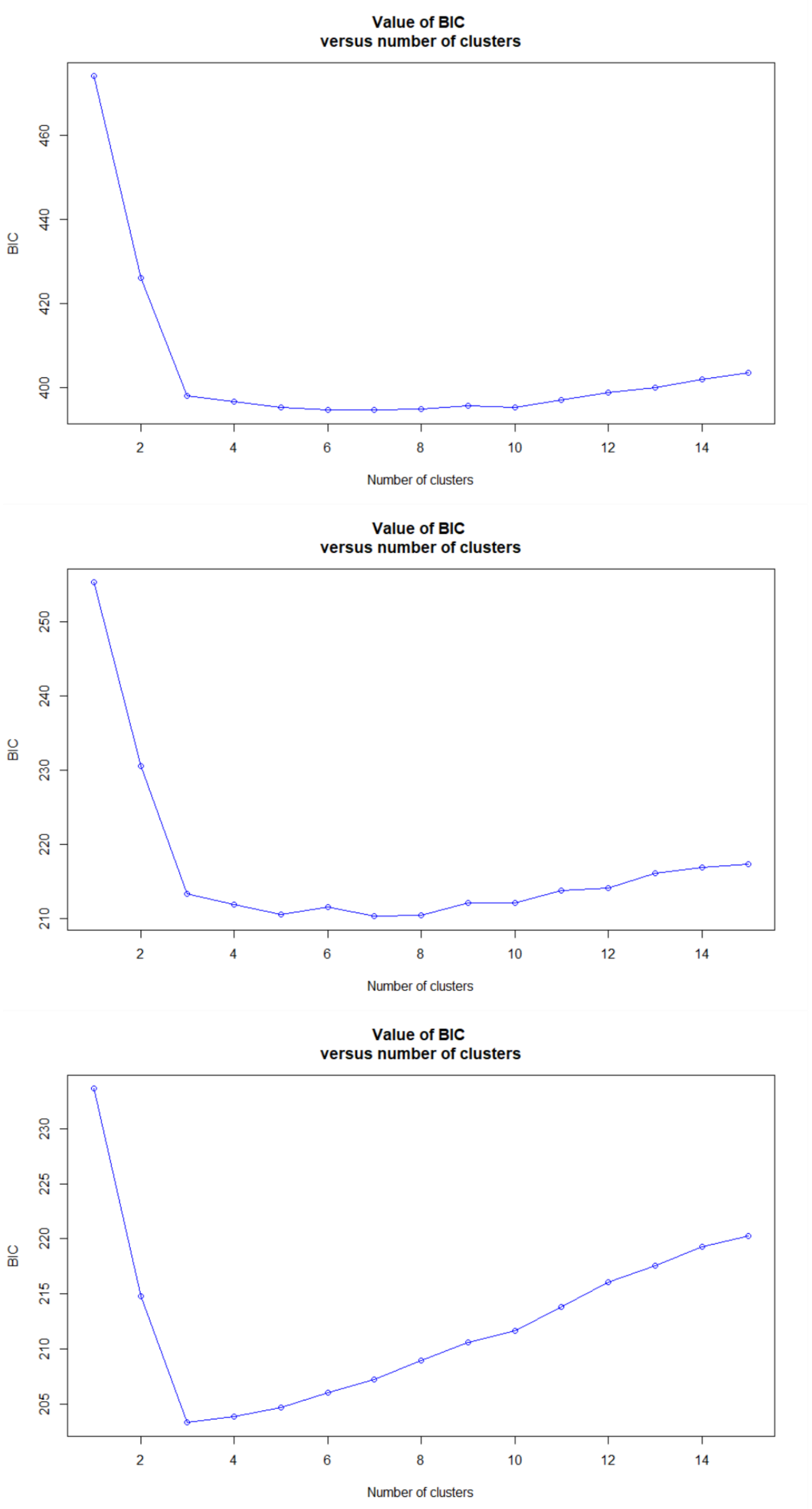

Fig. S3. Bayesian Information Criterion support for different K-values for DAPC in all samples combined (upper panel), contemporary samples only (middle panel), and resting eggs only (bottom panel).

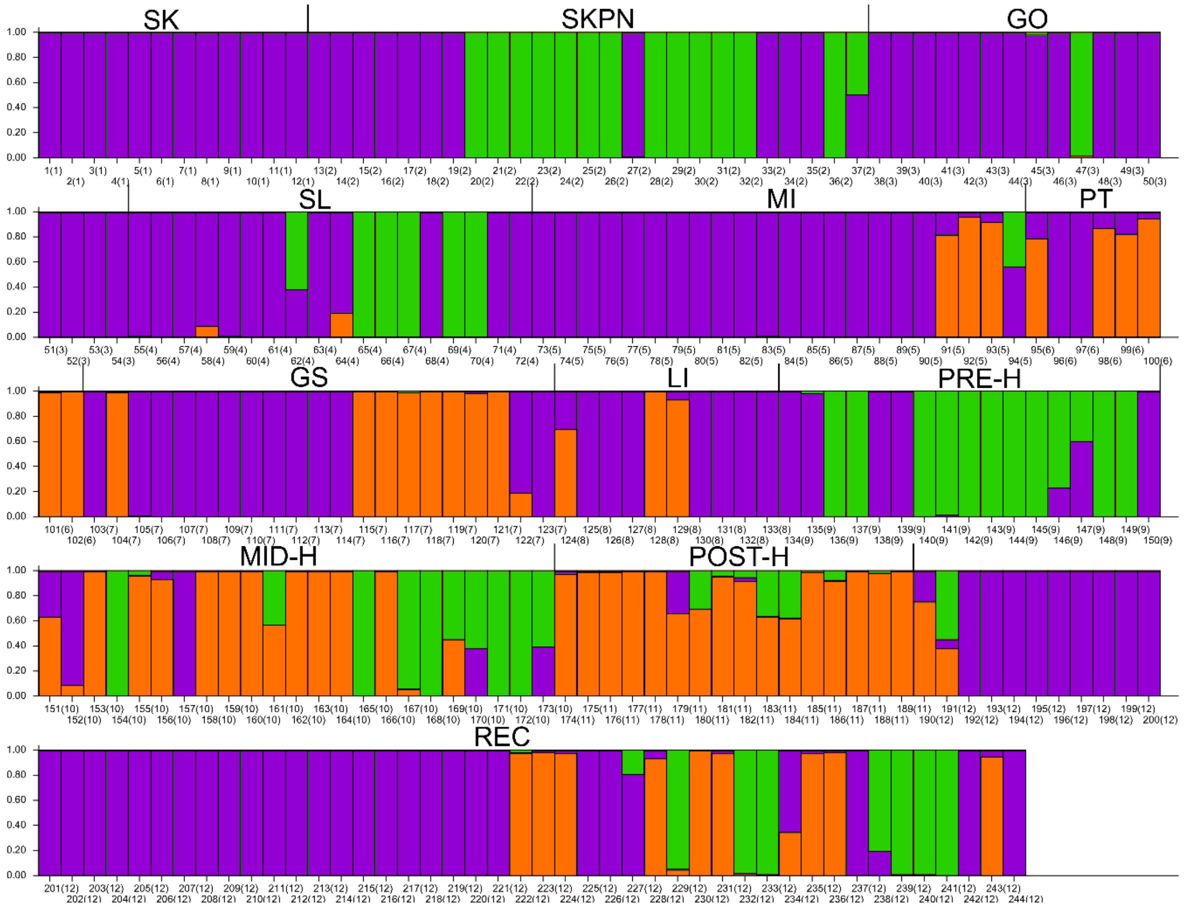

Fig. S4. Plot of the assignment probability of contemporary samples and resting eggs to three genetically distinct clusters (different colours) inferred with STRUCTURE, based on microsatellite data. Individuals are grouped by lake of origin. Each bar represents an individual's assignment probability to three inferred clusters (purple, green or orange). Contemporary samples are grouped as follows: three control lakes (SK, SKPN, GO), two transitional heated lakes (SL, MI) and three heated lakes (PT, GS, LI). Resting eggs are grouped as follows: PRE-H – produced before the onset of heating, MID-H – produced after the launch of the first plant but before the launch of the second power plant, POST-H – produced in the ca. 15 years following the launch of the second power plant, REC – produced recently, i.e. within 15 years of core collection.

Table S1. Basic information about investigated lakes.

Country	Lake	Abbrev.	Group	Longitude	Latitude	Altitude	Surface km^2	Max. depth m	Surf. Temp November*	Carlson's TSI(SD) November*
Poland	Skulskie	SK	Control	18°32′E	52°47′N	86	1.6	8	6.19	44.15
Poland	Gopło	GO	Control	18°38′E	52°50′N	77	21.5	17	6.55	61.93
Poland	Skulska Wieś	SKPN	Control	18°32′E	52°49′N	87	1.2	17	6.99	50.74
Poland	Gosławickie	GS	Heated	18°14′E	52°17′N	80	4.55	3	10.82	54.15
Poland	Licheńskie	LI	Heated	18°20′E	52°18′N	80	1.48	13	9.32	49.57
Poland	Pątnowskie	PT	Heated	18°17′E	52°18′N	79	2.83	5	7.62	47.52
Poland	Ślesińskie	SL	Heated	18°31′E	52°37′N	85	1.52	25	8	40.93
Poland	Mikorzyńskie	MI	Heated	18°31′E	52°35′N	85	2.52	37	9.82	40.93

* average of measurements in November 2012, 2014, 2015 and 2016
